# Architecting Highly Anisotropic Thermal Conductivity in Flexible Phase Change Materials for Directed Thermal Management of Cylindrical Li-Ion Batteries

**DOI:** 10.3390/ma18235400

**Published:** 2025-11-30

**Authors:** Liying Chen, Tong Yang, Jun Jiang, Jianwen Luo, Yuanyuan Li, Juntao Wang, Wanwan Li, Sujun Guan

**Affiliations:** 1 School of Civil Engineering, Henan University of Technology, Zhengzhou 450001, China; 2 Qingdao Haier Air Conditioner General Corp., Ltd., Qingdao 266101, China; 3 Qingdao Haier Smart Technology R&D Co., Ltd., Qingdao 266101, China; 4 School of Physics and Advanced Energy, R&D Center for Advanced Energy Materials, Henan University of Technology, Zhengzhou 450001, China

**Keywords:** li-ion battery thermal management, anisotropic thermal conductivity, phase change materials, hybrid carbon aerogel

## Abstract

**Highlights:**

**What are the main findings?**

- The hybrid 1D/3D carbon aerogel (CA) formed by combining 1D CNTs and 3D EG has a more optimized structural alignment than the single 3D EG-based CA as the CNTs enhance interlayer connectivity and guide directional pore arrangement;
- The hybrid 1D/3D-based FPCM demonstrated significantly enhanced anisotropic thermal conductivity: a 16.7% increase in axial thermal conductivity and a 5.0% increase in radial thermal conductivity compared to the single 3D-based FPCM;
- When applied to a cylindrical Li-ion battery, the hybrid FPCM effectively reduced the maximum battery-surface temperature by 13.1 °C and lowered the average surface temperature by 13.6 °C during the phase-change thermal-management stage.

**What are the implications of the main findings?**

- This work provides a scalable and effective strategy for designing high-performance anisotropic thermal-management materials, which can be tailored for high-power-density Li-ion batteries and other compact electronics with directional heat dissipation needs;
- The hybrid 1D/3D skeleton design overcomes the limitations of single-dimensional conductive networks, offering a new pathway to enhance thermal conductivity anisotropy beyond current upper limits;
- The developed FPCM maintains excellent flexibility, thermal stability, and energy storage capacity, making it suitable for practical applications in complex and flexible electronic devices.

**Abstract:**

The anisotropic jelly roll structure of cylindrical Li-ion batteries leads to highly directional heat generation, causing severe radial heat accumulation and creating a critical demand for precise thermal management. Conventional anisotropic phase change materials (PCMs), often reliant on single-dimensional conductive skeletons, exhibit limited enhancement in thermal conductivity anisotropy. This study proposes a novel strategy utilizing a hybrid carbon aerogel composed of one-dimensional carbon nanotubes (CNTs) and three-dimensional expanded graphite (EG) to construct highly aligned thermal conduction pathways within a flexible PCM. A three-step experimental method was employed to successfully fabricate a composite PCM with highly anisotropic thermal conductivity. A case study confirmed that, compared to a sole 3D skeleton, the hybrid 1D/3D aerogel significantly improves the alignment of the microstructure. At an optimal hybrid aerogel content of 8 wt.%, the composite achieved a 5.0% increase in radial thermal conductivity and a remarkable 16.7% increase in axial thermal conductivity, indicating a significantly optimized anisotropy ratio. When applied to a cylindrical battery thermal-management case, this material enables directed heat dissipation, effectively lowering the maximum battery-surface temperature by 13.1 °C. This work provides a scalable approach for designing high-performance anisotropic flexible PCMs tailored for advanced thermal management in high-power-density Li-ion batteries and other compact electronics.

## 1. Introduction

Li-ion batteries serve as the cornerstone energy storage technology for portable electronics and electric vehicles; however, their performance, cycle life, and safety are critically dependent on operating temperature [1,2]. During rapid charging and discharging, significant heat generation arises from internal ohmic resistance and electrochemical reactions. Compounding this issue, the prevalent jelly roll electrode configuration in cylindrical batteries leads to a highly anisotropic heat distribution, characterized by a radial thermal flux substantially exceeding the axial one [3,4]. This inhomogeneous heat generation readily causes localized hotspots and large temperature gradients, accelerating performance degradation and posing serious safety risks, including thermal runaway [5]. Consequently, developing efficient thermal-management strategies capable of precise, directional heat control tailored to this intrinsic anisotropic characteristic is imperative.

Conventional thermal-management techniques, such as air or liquid cooling, remain effective under moderate conditions [6,7]. Nevertheless, the continuous push for higher energy and power densities in Li-ion batteries results in dramatically increased internal heat flux and more pronounced anisotropic heat generation [8]. Traditional cooling methods, often relying on homogeneous materials, struggle to achieve the required precision in regulating heat flow paths, leading to limitations in either cooling efficiency or energy consumption. This existing condition provides the growing demand for advanced thermal-management technologies with directed heat-dissipation capabilities [9,10].

Thermal-management technology utilizing phase change materials (PCMs) demonstrates distinct advantages due to its ability to absorb heat efficiently and stabilize temperature through latent heat during phase transition [11,12,13]. A primary limitation, however, lies in the inherently low intrinsic thermal conductivity of most PCMs, which restricts the rate of heat dissipation. To address this, significant research efforts are directed toward developing composite PCMs by incorporating highly thermally conductive fillers [14]. A pivotal strategy within this domain involves the construction of an anisotropic thermal conductive skeleton within the PCM matrix. This approach focuses on creating a highly interconnected and oriented network. By infiltrating the molten PCM into this pre-designed skeleton, a continuous pathway for heat transfer is established, thereby significantly enhancing the overall thermal conductivity of the composite.

The effectiveness of this strategy is shown in several studies. For instance, Liang et al. [15] fabricated a graphene aerogel via freeze-drying and impregnated it with paraffin, achieving a composite PCM with a thermal conductivity of up to 2.7 W/(m·K)—nearly an order of magnitude higher than that of pure paraffin. Similarly, Shu et al. [16] employed a directional-freezing technique to produce a graphene aerogel with a vertically aligned architecture. After composite formation with paraffin, the material exhibited a high thermal conductivity of 4.36 W/(m·K), alongside pronounced anisotropic thermal transport properties. In another approach, Qiu et al. [17] utilized 3D printing to construct a scaffold with uniformly aligned carbon fibers. When combined with paraffin, the resulting composite reached a thermal conductivity of 2.89 W/(m·K), highlighting the considerable potential of engineered continuous skeletons in enhancing thermal conductivity.

These studies confirm that constructing an anisotropic thermal conductive skeleton for composite phase change materials is an effective strategy for achieving precise thermal management of the anisotropic heat generation in Li-ion batteries [18,19]. The validity of this approach has been corroborated by several recent investigations. For instance, Wei et al. [20] developed an anisotropic composite PCM based on an aligned graphite nanoplatelet skeleton for mobile-phone-chip thermal management. Under a high heat flux of 5 W/cm^2^, this material significantly reduced the chip’s hotspot temperature by approximately 22 °C. Similarly, Zandieh et al. [21] applied a composite PCM with aligned boron nitride nanotubes to the thermal management of a cylindrical Li-ion battery. At a 3C discharge rate, it successfully maintained the maximum battery-surface temperature below 40 °C, outperforming conventional homogeneous materials by a temperature reduction exceeding 10 °C. These cases demonstrate that utilizing anisotropic PCMs for directed thermal management is a viable pathway to enhance heat dissipation in high-power electronic devices.

Although the introduction of single-dimensional skeletons (e.g., 1D carbon nanotubes, 2D graphene, or 3D metal foams) can impart a certain degree of anisotropy to PCMs, their anisotropy ratio (typically <5) often proves insufficient for the extreme heat dissipation demands of next-generation ultra-high-power-density Li-ion batteries [22,23,24]. Researchers have widely employed 1D materials (e.g., CNTs and SiC nanowires) to construct axially aligned channel structures via external field alignment or ice-templating methods [25,26]. Alternatively, 2D materials (e.g., graphene and BN nanosheets) have been used to form in-plane-oriented layered skeletons through layer-by-layer assembly or directional freezing [27,28]. Intrinsically isotropic 3D materials (e.g., metal foams and carbon sponges) have also been utilized [29,30]. These methods, based on single-dimensional precursors, have successfully established preliminary anisotropic thermal pathways within the PCM matrix. For example, Li et al. [31] prepared an aerogel skeleton via directional freezing of a CNT suspension, achieving an axial thermal conductivity (~1.05 W/m·K) vastly superior to its radial conductivity (~0.38 W/m·K), with an anisotropy ratio of about 2.76. Guo et al. [32] obtained a graphene aerogel with an axial-to-radial thermal conductivity ratio of up to 4.2 using a similar method. Despite these significant advances, the anisotropy ratios achievable with single-dimensional skeletons show limitations in the face of the stringent thermal requirements of advanced electronics. Therefore, exploring new skeleton construction strategies to surpass the current upper limit of anisotropic thermal conductivity remains a critical scientific challenge [33,34,35,36].

Recently, hybridizing nanomaterials of different dimensions to synergistically enhance material properties has gained considerable attention [37,38]. Theoretical studies suggest that incorporating one-dimensional nanomaterials as "bridges" within a three-dimensional macroscopic skeleton can optimize the orientation and connectivity of the thermal network, thereby significantly enhancing thermal conductivity in a specific direction.

Inspired by this, this study proposes a novel hybrid carbon aerogel thermal skeleton fabricated by combining one-dimensional carbon nanotubes (CNTs) with three-dimensional expanded graphite (EG). It is hypothesized that the rigid 3D EG framework can serve as a primary support structure, while the high-aspect-ratio 1D CNTs act as directional guides and enhancers of interlayer connectivity, working synergistically to create a highly ordered anisotropic thermal pathway. This study will elaborate on the preparation of this hybrid aerogel and its corresponding flexible composite PCM (FPCM), systematically characterize its thermophysical properties (with emphasis on its highly anisotropic thermal conductivity), and finally, evaluate its practical application efficacy using a cylindrical Li-ion battery as a case study for directed thermal management. The aim is to provide new insights and a practical foundation for developing high-precision thermal-management solutions tailored for high-power-density energy storage devices.

## 2. Materials and Methods

### 2.1. Materials

**Figure 1:**
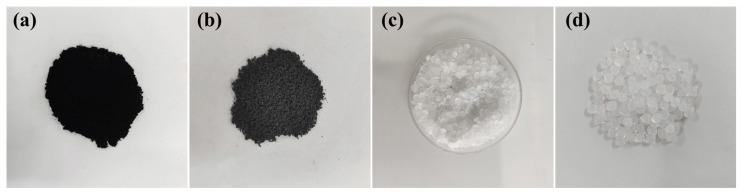
The main raw materials for fabricating hybrid 1D/3D-based FPCM: (**a**) CNTs, (**b**) EG, (**c**) PA, (**d**) OBC.

EG (particle size: 50 mesh, expansion ratio: 600 mL/g) served as the 3D precursor for fabricating CA with aligned networks of high thermal conductivity. CNTs (tube diameter: 3–15 nm, bulk density: 0.06 g/cm^3^) acted as the 1D precursor for constructing hybrid CA. A synergistically assembled CNT/EG hybrid CA with enhanced anisotropy was employed as the thermal conductive skeleton. Paraffin (PA, melting point: 58 °C, latent heat: 210 kJ/kg) functioned as the phase-change matrix. Olefin block copolymer (OBC; melting point: 110 °C, density: 0.88 g/cm^3^) was selected as the flexible carrier to encapsulate and structurally stabilize the PCM while imparting structural flexibility to the composite for integration into electronics with complex geometries. Calcium chloride solution (CaCl_2_, 0.20 mol/L) served as an alignment-promoting modifier for the vertically oriented carbon skeleton. Based on prior optimization studies [39], the three-component composite of CA, PA, and OBC was formulated at a mass ratio of 8:60:32. The main raw materials are shown in Figure 1.

### 2.2. Preparation of 1D/3D Hybrid CA

**Figure 2:**
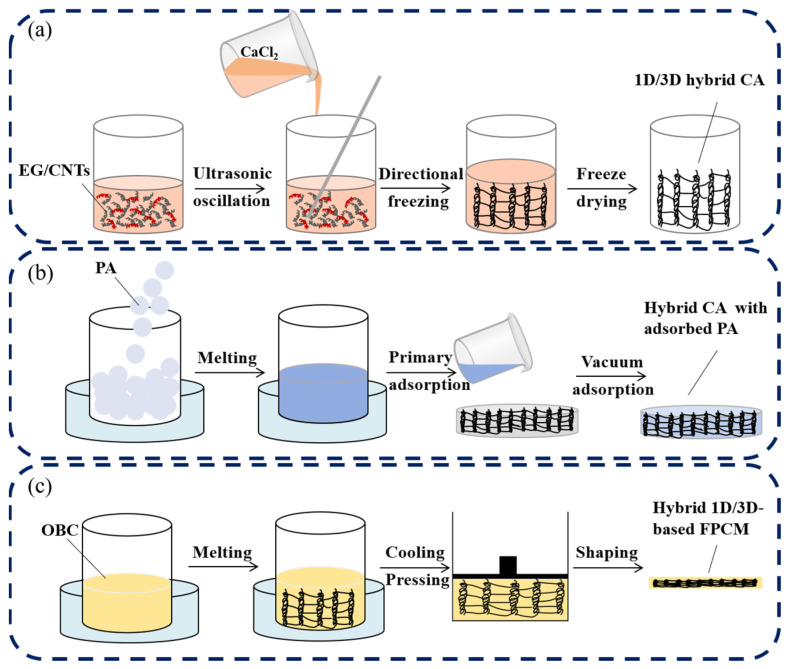
Preparation flowchart of (**a**) 1D/3D hybrid CA, (**b**) hybrid PA-adsorbed CA, and (**c**) hybrid 1D/3D-based FPCM.

The hybrid EG/CNT CA with enhanced anisotropy was fabricated via a directional freezing method using 1D CNTs and 3D EG as precursors. EG and CNTs at a mass ratio of 1:1 were mixed with aqueous solution in a 30:1:1 proportion and ultrasonically dispersed for 30 min to form a homogeneous carbon-based suspension. Subsequently, CaCl_2_ solution was added and agitated for 10 min to strengthen the cross-linking interactions between carbon layers. The mixture was then directionally frozen in a low-temperature vacuum chamber (−50 °C, 15 Pa). The hybrid solution was placed in a cryogenic-resistant glass vial, with thermally insulating materials arranged along its lateral surface to establish a unidirectional temperature gradient imposed by the directional freezing apparatus. This configuration constrained cryogenic conditions to the axial direction of the vial, inducing preferential ice crystal formation along the axial temperature gradient. Consequently, the EG framework undergoes oriented alignment along the ice crystal growth direction, while fibrous CNTs exhibit axial distribution under the mechanical constraint of ice crystal propagation. This axial arrangement of carbon atoms within the CNT lattice further promotes ordered structural alignment of the EG carbon framework, enhancing the overall anisotropy of the composite architecture. Following complete ice crystallization, the sample was freeze-dried for 72 h under vacuum to sublimate ice crystals, leaving a porous carbon skeleton with high anisotropy and adsorption capacity. This process preserved the ice-templated voids as aligned channels, resulting in a hierarchically structured hybrid CA. The preparation process is illustrated in Figure 2a.

### 2.3. Preparation of Hybrid 1D/3D-Based FPCM

The prepared 1D/3D hybrid CA was placed in a high-temperature-resistant glass vessel. Molten PA was cast into the vessel (Figure 2b) and subjected to vacuum-assisted impregnation (80 °C, 0.04 MPa, 1 h) to ensure complete PA infiltration into the CA skeleton. Subsequently, the molten OBC was introduced into the aforementioned glass vial containing the CA-PA composite (Figure 2c), and the mixture was subjected to vacuum infiltration in an oven maintained at 80 °C, enabling complete encapsulation of the CA and PA framework by the molten OBC matrix. After thorough mixing of the three components, the mixture was placed at room temperature to allow for natural cooling of the composite. Moderate pressure was mechanically pressurized during solidification, yielding the FPCM with an interconnected anisotropic thermal conductive network.

### 2.4. Characterizations

The microstructural morphology was examined using scanning electron microscopy (SEM, ZEISS Sigma 300, Oberkochen, Germany). The phase-change temperature and latent heat of FPCMs were determined by differential scanning calorimetry (DSC, Q20, New Castle, DE, USA; uncertainty: 1.0%, temperature accuracy: ±2.0 °C). Axial and radial thermal conductivities were measured via the transient plane-source method (TPS, Hot Disk, Gothenburg, Sweden; uncertainty: 2.0%). The chemical compositions of all materials were analyzed by X-ray diffraction (XRD, SmartLab SE, Tokyo, Japan) with a scanning angle range of 10–50° and scanning rate of 2°/min. Additionally, thermal stability and flexibility evaluations were conducted to explore the fundamental thermal properties of hybrid 1D/3D-based FPCMs.

## 3. Results

### 3.1. Microstructural Morphology Analysis

**Figure 3:**
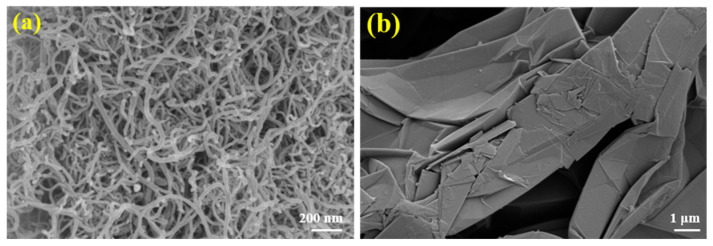
Microstructural morphology of (**a**) initial CNTs and (**b**) initial EG.

**Figure 4:**
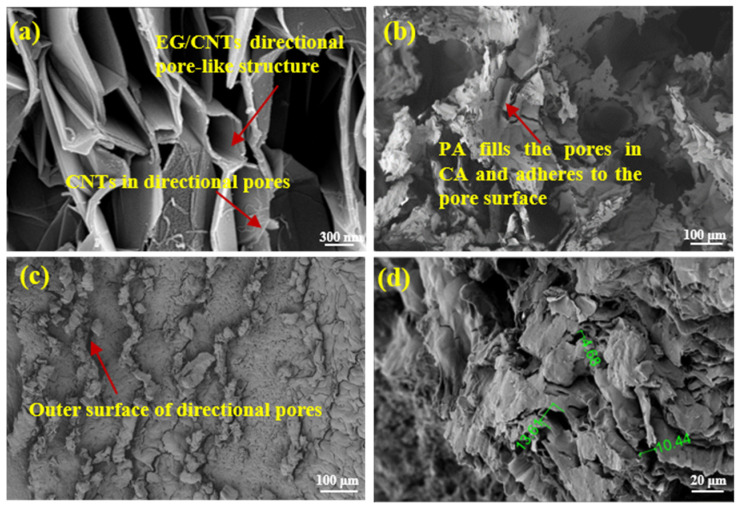
Microstructural morphology of 1D/3D hybrid CA and FPCMs: (**a**) Hybrid CA, (**b**) hybrid PA-adsorbed CA, (**c**) external surface of hybrid 1D/3D-based FPCM, (**d**) internal section of hybrid 1D/3D-based FPCM.

**Figure 5:**
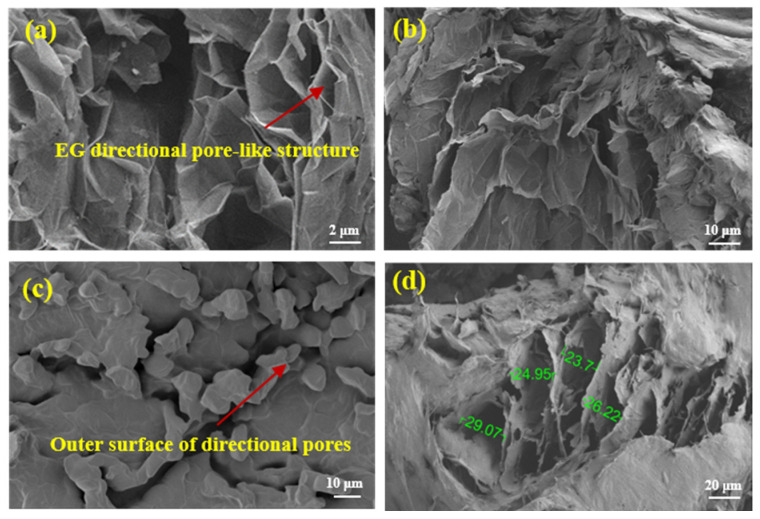
Microstructural morphology images of single 3D-based CA and the prepared FPCMs: (**a**) Single 3D-based CA, (**b**) single 3D-based PA-adsorbed CA, (**c**) external surface of single 3D-based FPCM, (**d**) internal section of single 3D-based FPCM.

As shown in Figure 3, the microstructures of pristine EG and CNTs present a disordered arrangement, which is unfavorable for efficient thermal conduction. The microstructural morphologies of the 1D/3D hybrid CA and the resulting FPCMs fabricated with this CA as the thermal conductive skeleton are illustrated in Figure 4. The hybrid CA features well-aligned pore structures, with 1D CNTs distributed both on the 3D EG carbon lamellae and within their axial pores (Figure 4a). The CNTs distributed on the carbon lamellae establish thermal conduction pathways between adjacent layers, while their fibrous morphology guides the directional alignment of pores within the axial direction of EG, thereby enhancing structural anisotropy. Comparative analysis with the single 3D-based CA morphology in Figure 5a confirms superior structural alignment in the hybrid CA. This synergy leverages the intrinsic alignment capability of 1D CNTs and the bulk interconnectivity of 3D EG, yielding a CA skeleton with enhanced directional ordering.

Figure 4b shows the morphology of 1D/3D hybrid CA after adsorption of PA. The PA is uniformly infiltrated into the aligned pores of the CA without structural collapse. Upon encapsulation by molten OBC, a highly compatible three-component composite is formed. As illustrated in Figure 4c, the OBC coating exhibits an aligned surface morphology consistent with the pore orientation of the hybrid CA, confirming that the encapsulation process preserves the directional architecture of the CA skeleton. Cross-sectional analysis (Figure 4d) further reveals that the interconnected aligned skeleton of the CA remains intact within the three-component composite, with no structural degradation induced by OBC encapsulation. These results validate the effectiveness of our methodology in achieving integration of the hybrid CA skeleton with the phase-change matrix and flexible carrier, successfully fabricating anisotropic FPCMs with structurally preserved directional thermal conductive networks.

Comparative analysis of Figure 4 and Figure 5 reveals pronounced differences in microstructural orientation between single 3D-based CA and CNT/EG hybrid CA. Whereas the hybrid CA exhibits aligned pore channels with CNTs distributed across EG layers and axial pores (Figure 4a), the single 3D-based CA shows no CNT presence and manifests significantly higher non-parallelism in pore channel axes, accompanied by coarser porous architecture. Although both systems demonstrate effective PA infiltration through oriented pores (Figure 4b and Figure 5b), the hybrid 1D/3D-based FPCM exhibits more ordered PA alignment. Notably, the hybrid system’s exterior morphology manifests superior anisotropy (Figure 4c), with its internal thermally conductive network exhibiting finer structure compared to the single 3D-based FPCM (Figure 4d). This structural disparity enables more ordered accommodation of PA and OBC within the directional framework of hybrid 1D/3D-based FPCMs, which likely constitutes the critical structural determinant accounting for the observed anisotropy in thermal conductivity between these composite systems.

### 3.2. Chemical Composition Analysis

**Figure 6:**
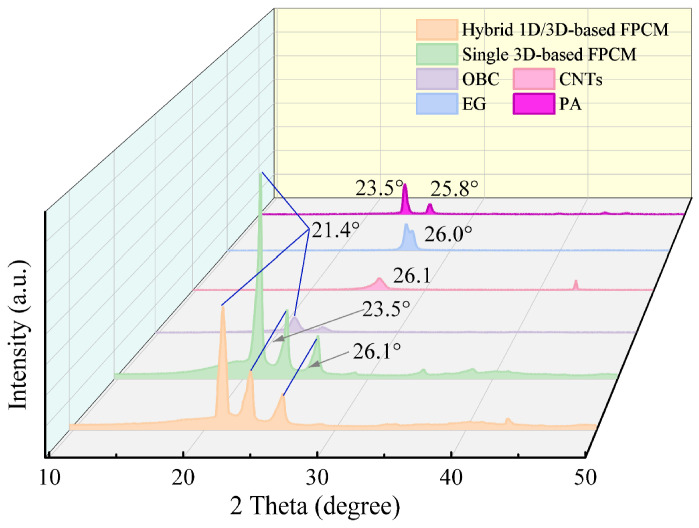
XRD patterns of PA, EG, CNTs, OBC, and FPCM.

The XRD patterns of anisotropic FPCMs are presented in Figure 6. PA exhibits diffraction peaks at 23.50° and 25.86°, while the EG and CNTs show characteristic peaks at 26.08° and 26.10°, respectively. The diffraction peak of OBC appears at 21.34°. Both single CA-based and hybrid 1D/3D-based FPCMs prepared from the three components exhibit diffraction peaks that precisely match those of PA, EG, CNTs, and OBC. Furthermore, no emergence of new peaks or disappearance of original peaks is observed. This confirms that the interactions between CA, PA, and OBC are purely physical, with no chemical reactions occurring during composite formation, and that the three-dimensional framework is a result of physical assembly and ice-templating. These results indicate that the hybrid 1D/3D-based FPCM preserves the intrinsic properties of the phase-change matrix PA, ensuring stable thermal energy storage functionality.

### 3.3. Phase-Change Property Analysis

**Figure 7:**
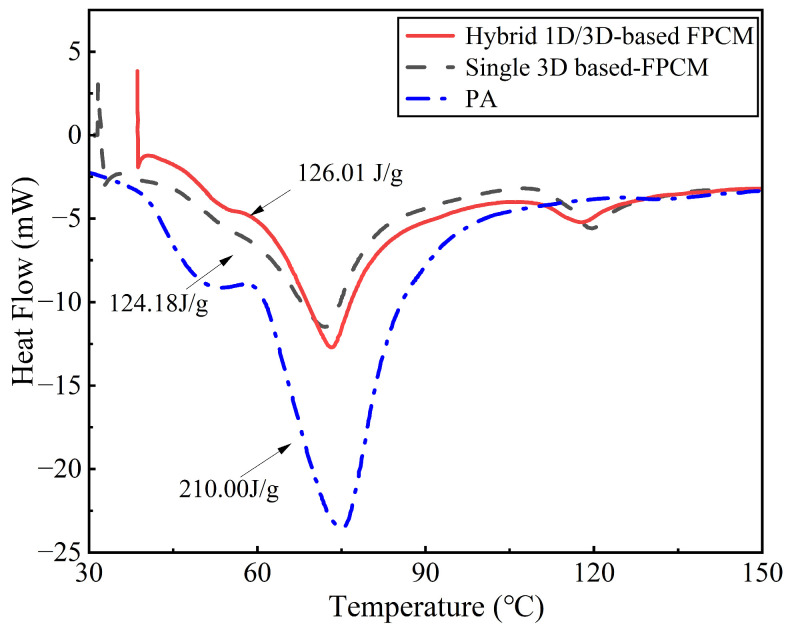
DSC curves of PA, hybrid 1D/3D-based FPCM, and single 3D-based FPCM.

To investigate whether the phase-change characteristics of anisotropic FPCMs are influenced by CA alignment, the phase-change parameters of FPCMs fabricated from hybrid CA and a single 3D-based CA with identical component ratios were compared, as shown in Figure 7. Both FPCMs undergo phase change within the range of 60–70 °C, which is consistent with the phase-change range of the PA matrix. The latent heat values of the two FPCMs, summarized in Table 1, remain high (~120 J/g), corresponding to ~57% of the latent heat of the PA (210 kJ/kg). This result is in good agreement with the mass fraction of PA (55.6%) in the three-component composite. The slight deviation from the theoretical value is due to a modified potential energy state of the PA molecules within the composite, which restricts their phase transition behavior. Despite this molecular-level effect, the result confirms that the energy storage capacity is not compromised by the enhanced anisotropy and remains predominantly governed by the PA content.

### 3.4. Thermal Conductivity Analysis

**Figure 8:**
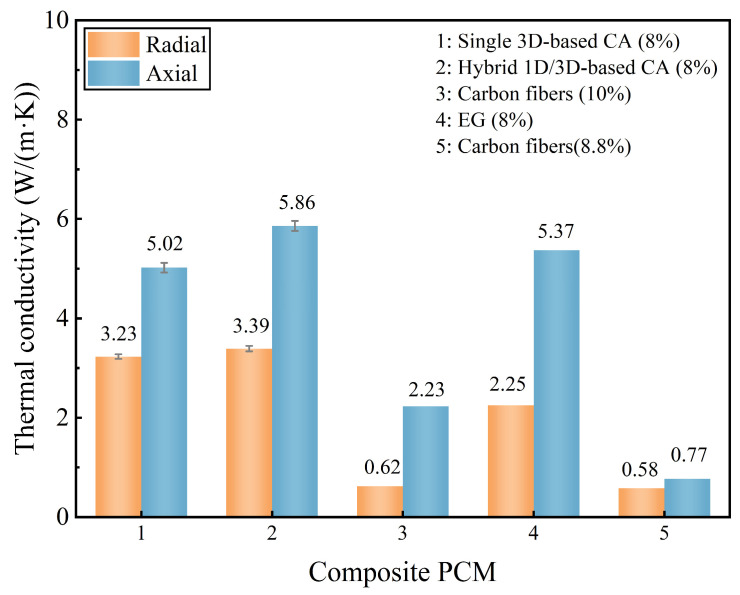
Thermal conductivity comparison of composite PCMs with different thermal conductive enhancers (content indicated in top-right insets, data for composite PCMs 1, 2 are mean ± SEM (n = 5), data for composite PCMs 3–5 are from references [40,41,42]).

To elucidate the impact of structural anisotropy on the bidirectional thermal conductivity of FPCM, a comparative analysis is conducted between the newly prepared hybrid 1D/3D-based FPCM and the previously reported single 3D-based FPCM. Figure 8 compares the axial and radial thermal conductivities of the fabricated composite materials, presenting mean values derived from five independent measurements. The axial thermal conductivity of the FPCM prepared with 1D/3D hybrid CA reaches 5.86 W/(m·K), which is 0.84 W/(m·K) higher than the axial thermal conductivity of the single 3D-based FPCM, with an enhancement of about 16.7%. This is because the internal high-thermal-conductivity enhancers form a highly oriented arrangement structure in the axial direction after directional freezing; the high-thermal-conductivity carbon lamellae of hybrid CA has a higher degree of regularity at the microscopic level; and the fibrous structure of the CNTs themselves can promote the continuous lapping between the sheet layers to a certain extent, so the heat can be transferred to the outside world efficiently in this direction. Meanwhile, its radial thermal conductivity is 3.39 W/(m·K), and this value is enhanced by 5.0% compared with that of a single 3D-based FPCM. This indicates that the CNTs in the hybrid CA, filled in the EG sheet layer and between the pores, also play an active role in the heat transfer in this direction.

In addition, comparing the FPCMs prepared in this study with some of the currently published anisotropic composite PCMs [40,41,42], it can be found that the FPCMs prepared with hybrid CA, with the same or even a lower content of thermal conductivity enhancers, have higher thermal conductivities. In summary, the hybrid 1D/3D-based FPCMs not only exhibit obvious anisotropic features in thermal conductivity, but their unique micro-morphology also exacerbates the anisotropy in thermal conductivity distribution. This characteristic makes this type of FPCM more adaptable to the urgent need for efficient anisotropic heat dissipation in localized-heat-source electronic devices.

### 3.5. Thermal Stability Analysis

**Figure 9:**
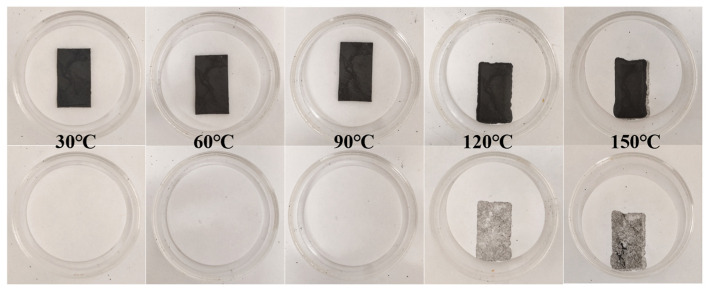
Anti-leakage testing of hybrid 1D/3D-based FPCM.

**Figure 10:**
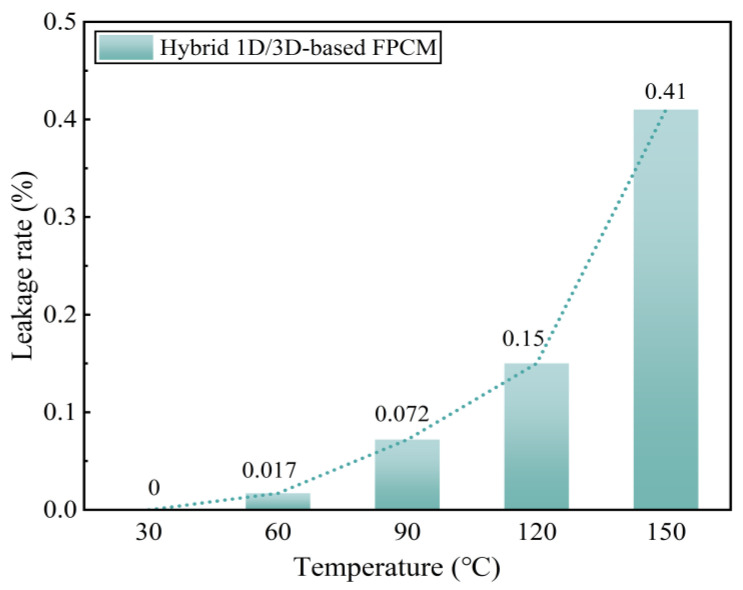
Mass change rate of hybrid 1D/3D-based FPCM at various temperatures.

To investigate the potential impact of anisotropic enhancement induced by hybrid CA on the anti-leakage properties of FPCMs, the morphological stability (Figure 9) and mass leakage rate (Figure 10) of the newly prepared hybrid 1D/3D-based FPCM were systematically analyzed after 1 h heating over a temperature range of 30–150 °C. As revealed in Figure 9, the hybrid 1D/3D-based FPCM exhibits excellent shape stability below 90 °C, with negligible phase-change matrix leakage observed. Consistently, Figure 10 indicates a mere 0.072% mass leakage at 90 °C. However, when the temperature surpasses 90 °C, the leakage traces on filter paper progressively intensified. The main reason for this phenomenon is that the softening temperature of the flexible support carrier OBC is 113 °C; when the temperature exceeds 90 °C or is close to 113 °C, the OBC begins to soften, and its encapsulation and shaping effect on the PA is subsequently weakened. With further temperature elevation, OBC approaches melting transition, enabling fully phase-changed PA to migrate more readily to the material surface, resulting in leakage. Notably, this leakage behavior aligns with the anti-leakage characteristics of a single 3D-based FPCM reported in the literature [36]. In conclusion, the incorporation of hybrid CA does not compromise the anti-leakage performance of FPCMs, as the hybrid system maintains superior leakage resistance below 90 °C.

### 3.6. Flexibility Analysis

**Figure 11:**
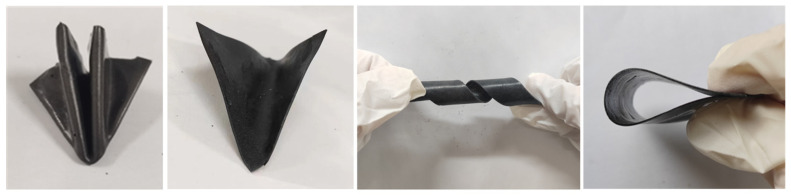
Flexibility testing of hybrid 1D/3D-based FPCM.

The introduction of a hybrid CA significantly enhances the anisotropic properties of FPCMs. In order to systematically evaluate whether this enhancement has any effect on the flexibility of FPCMs, the newly prepared hybrid 1D/3D-based FPCM is tested in a variety of morphologies—including folding and bending—in this study, and the test results are shown in Figure 11. It should be emphasized that the role of the OBC (with an intrinsic thermal conductivity of ~0.54 W/(m·K)) is primarily to serve as a flexible supporting matrix—providing encapsulation and shape stability—rather than as a primary contributor to the skeletal structure or the overall thermal conductivity.

At room temperature, the newly prepared FPCM exhibits exceptional flexibility, withstanding folding and twisting without brittle fracture under external forces. This superior flexibility originates from the unique architecture of the OBC, which consists of alternating crystalline hard segments and amorphous soft segments. The hard segments maintain the polymer’s long-chain stability, while the soft segments—composed of highly mobile, elastic molecular chains—endow the composite with excellent foldability and bendability. Notably, when the OBC is complexed with the hybrid CA and the phase-change matrix, the flexibility properties of its soft block are retained so that the newly prepared three-component blends still exhibit excellent flexibility, which is similar to the flexibility characteristics of the single 3D-based FPCM. These results confirm that the hybrid CA enhances anisotropy without sacrificing flexibility, making it highly suitable for anisotropic thermal management in electronics with complex geometries.

### 3.7. Anisotropic Thermal-Management Capability Analysis

**Figure 12:**
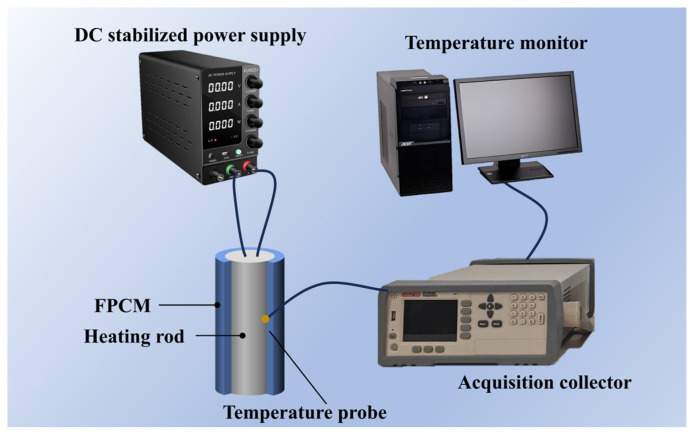
Experimental test system for thermal-management performance.

To analyze the thermal-management capability of the hybrid 1D/3D-based FPCM, the prepared FPCM was wrapped around the surface of a cylindrical heating rod (diameter 18 mm, height 65 mm) to simulate the heat generation of an 18650-type Li-ion battery (rated capacity: 2200 mAh, internal resistance: 65 mΩ) during the discharge process. For cylindrical Li-ion batteries, heat generation is predominantly derived from internal resistance heat, while the proportions of chemical reaction heat, polarization heat, and secondary reaction heat in total battery heat generation are extremely small and, thus, negligible. Therefore, only internal resistance heat was considered in the battery-heat-generation simulation of this study. At a 4C discharge rate, the total heat-generation power Q was 5 W, corresponding to a heat flux density of approximately 1361 W/m^2^. The specific thermal-management test system is shown in Figure 12. A DC power supply was used to provide a fixed power of 5 W to the heating rod, and the heating rod converted the electrical power into heat through resistance wires. An Amber multi-channel temperature measuring instrument was employed to test the surface temperature rise in the benchmark battery (simulated by the heating rod).

**Figure 13:**
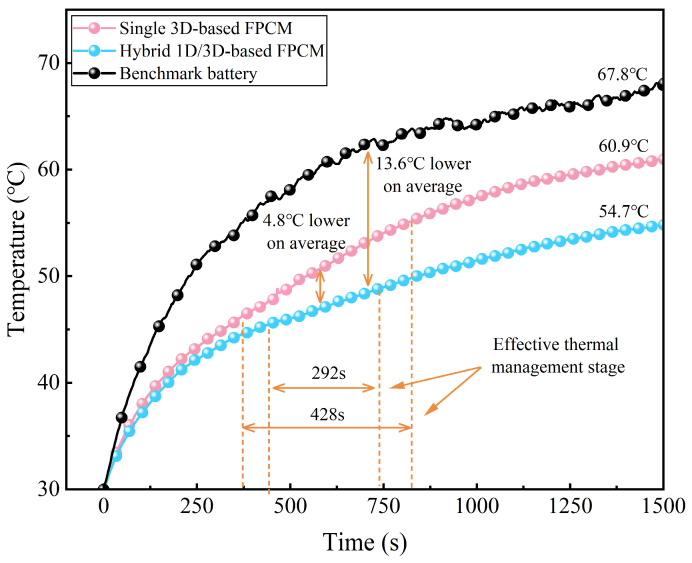
Curves of battery-surface temperature rise without any material, with single 3D-based FPCM, and with hybrid 1D/3D-based FPCM.

The temperature-rise data of the battery surface under three conditions—without any thermal-management material, with a single 3D-based FPCM, and with a hybrid 1D/3D-based FPCM—are presented in Figure 13. The results indicate that the introduction of FPCMs significantly suppresses the surface temperature of the battery. Both types of FPCMs exhibit distinct phase-change endothermic stages, with durations of 428 s and 292 s, respectively. These durations represent the effective thermal-management stage, defined as the period from when the battery-surface temperature reaches the onset of the FPCM’s phase change to the point where the latent heat is substantially depleted and the temperature begins to rise again. The shorter duration for the hybrid FPCM (292 s) compared to the single 3D-based FPCM (428 s) is primarily attributed to its higher axial thermal conductivity, which enables more efficient heat spreading and a faster transition through the phase-change plateau under the same heating power. 

Notably, during the phase-change thermal-management stage, the average temperature of the battery under the action of the hybrid 1D/3D-based FPCM is 4.8 °C lower than that with the single 3D-based FPCM. This phenomenon is primarily attributed to the improved axial thermal conductivity of the hybrid 1D/3D-based FPCM, a characteristic that facilitates the rapid transfer of heat from the radial surface of the battery to the external environment, thereby significantly enhancing the thermal-management efficiency of the PCM. Compared with the benchmark battery without any thermal-management material, the hybrid 1D/3D-based FPCM reduces the average surface temperature by 13.6 °C during this stage. In addition, when the system finally reaches thermal equilibrium, the temperatures of the batteries using the two types of FPCMs are 13.1 °C and 6.9 °C lower than that of the benchmark battery, respectively. These comparative results demonstrate that the hybrid 1D/3D-based FPCM is more capable of meeting the thermal-management requirements of cylindrical Li-ion batteries.

## 4. Conclusions

To address the challenge of directed thermal management for Li-ion batteries with intrinsic anisotropic heat generation, this study successfully architected a highly aligned 1D/3D hybrid CA through directional freezing. By employing vacuum impregnation and melt blending, a corresponding flexible composite phase change material (FPCM) was fabricated, integrating high-directional thermal conductivity with excellent flexibility. Performance characterization confirmed the following key findings:

(1) The hybrid CA synergistically combines the robust 3D framework of expanded graphite with the high orientation capability of 1D carbon nanotubes, yielding a thermally conductive skeleton with superior structural alignment compared to CA derived solely from EG.
(2) Owing to the optimized skeleton, the resulting FPCM exhibits a substantially enhanced thermal conductivity anisotropy. Specifically, compared to its 3D CA-based counterpart, the radial thermal conductivity increased by 5.0%, while the axial thermal conductivity was significantly improved by 16.7%.
(3) The hybrid 1D/3D-based FPCM maintains considerable latent heat capacity, excellent shape stability, and superior flexibility, ensuring reliable performance under practical conditions.
(4) When applied to cylindrical Li-ion batteries, the material demonstrates exceptional effectiveness in directed thermal management. It achieved a substantial reduction in the battery’s average surface temperature by 13.6 °C during the phase-change thermal-management stage, effectively mitigating localized overheating.

This work validates the strategic design of hybrid conductive skeletons for developing high-performance anisotropic FPCMs, providing an efficient and targeted thermal-management solution for advanced Li-ion batteries with pronounced anisotropic heat-generation characteristics. Future work will focus on applying this material to commercial 18650 cylindrical battery systems and evaluating critical safety aspects to facilitate its eventual real-world application.

## Figures and Tables

**Table 1:** Phase-change parameters of hybrid 1D/3D-based FPCM and single 3D-based FPCM.

Sample	CA Content (%)	Phase-Change Temperature (°C)	Latent Heat (J/g)
Hybrid 1D/3D-based FPCM	8	60.9	126.01
Single 3D-based FPCM	8	60.1	122.18

## Data Availability

The original contributions presented in this study are included in the article. Further inquiries can be directed to the corresponding authors.
